# Characterization of the Testis-Specific Angiotensin Converting Enzyme (tACE)-Interactome during Bovine Sperm Capacitation

**DOI:** 10.3390/cimb44010031

**Published:** 2022-01-17

**Authors:** Mina Ojaghi, Jacob Varghese, John P. Kastelic, Jacob C. Thundathil

**Affiliations:** Department of Production Animal Health, Faculty of Veterinary Medicine, University of Calgary, Calgary, AB T2N 4N1, Canada; mina.ojaghi@ucalgary.ca (M.O.); jacob.varghese@ucalgary.ca (J.V.); jpkastel@ucalgary.ca (J.P.K.)

**Keywords:** bovine, spermatozoa, capacitation, interactome, tACE

## Abstract

A comprehensive understanding of molecular and biochemical changes during sperm capacitation is critical to the success of assisted reproductive technologies. We reported involvement of the testis-specific isoform of Angiotensin Converting Enzyme (tACE) in bovine sperm capacitation. The objective of this study was to characterize the tACE interactome in fresh and heparin-capacitated bovine sperm through immunoprecipitation coupled with mass spectrometry. These interactions were validated by co-localization of tACE with beta-tubulin as an identified interactome constituent. Although interactions between tACE and several proteins remained unchanged in fresh and capacitated sperm, mitochondrial aldehyde dehydrogenase 2 (ALDH2), inactive serine/threonine protein-kinase 3 (VRK3), tubulin-beta-4B chain (TUBB4B), and tubulin-alpha-8 chain (TUBA8) were recruited during capacitation, with implications for cytoskeletal and membrane reorganization, vesicle-mediated transport, GTP-binding, and redox regulation. A proposed tACE interactional network with identified interactome constituents was generated. Despite tACE function being integral to capacitation, the relevance of interactions with its binding partners during capacitation and subsequent events leading to fertilization remains to be elucidated.

## 1. Introduction

Capacitation comprises a series of structural and functional modifications normally occurring in sperm in the female reproductive tract, enabling fertilizing ability [1,2,3]. Capacitated sperm are characterized by an increased content of tyrosine-phosphorylated proteins and intracellular secondary messenger molecules, greater plasma membrane fluidity due to cholesterol efflux, and hyperactivated motility [4,5,6]. Sperm are traditionally regarded as terminally differentiated cells lacking transcriptional and translational activity, with cellular functions predominantly regulated by proteins already present in sperm through post-translational modifications [7]. However, recent studies posit de novo protein synthesis through translation on mitochondrial ribosomes in bovine, murine, human, and boar sperm [8,9,10], alluding to the functional significance of the dynamic sperm proteome to reproductive outcomes. Therefore, elucidation of the role of specific sperm proteins and their interactions and localization during capacitation is essential for a comprehensive understanding of the regulation of sperm function.

The testis-specific isoform of Angiotensin Converting Enzyme (tACE) is an isozyme of somatic Angiotensin Converting Enzyme (sACE) implicated in the regulation of sperm capacitation, acrosome reaction, and zona pellucida binding [11,12,13]. Furthermore, we recently reported that the content and activity of tACE in frozen-thawed bull sperm were associated with in vivo fertility [13,14,15]. Sperm from knockout mice lacking tACE had reduced fertility due to defective sperm transport and impaired oocyte binding [16,17], which was not rescued with isozymic substitution of sACE [18], demonstrating the in vivo functional difference between these two isoforms. Structurally, the ~110 kDa tACE contains only a single N-terminal catalytic active site domain and is smaller in molecular weight relative to the ~150 kDa somatic isoform of the enzyme with a consensus HEXXH zinc-binding motif in the enzymatic active site shared between both isozymes [19]. In addition to its reported male fertility-contingent dicarboxypeptidase activity [11], tACE may also enzymatically function through glycosylphosphatidylinositol (GPI)-anchored protein releasing (GPIase) activity, resulting in shedding of GPI-anchored proteins in vivo [12]. These two independently regulated enzymatic functions are regarded as necessary for sperm capacitation and fertilization [12]. However, the presence and functionality of GPIase in tACE is disputed [20], despite tACE enzymatic activity reportedly being essential for male fertility [11].

Characterization of the spatial localization of tACE and its interactome during capacitation would advance current knowledge regarding the molecular regulation of this process. We previously reported that bovine tACE is selectively expressed in the acrosomal region and principal piece of fresh sperm and is subsequently localized to the post-acrosomal region in heparin-capacitated sperm [13,14]. Other investigations have also established that various phosphotyrosine-containing proteins such as heat-shock protein [21] and ATP1A4 [22] undertake a comparable redistribution to the post-acrosomal region during capacitation. However, it remains to be determined whether these proteins are newly synthesized through mitochondrial translational machinery [9] and compartmentalized at specific subcellular locations, or if existing proteins are spatially re-localized to these regions. Although the mechanisms and dynamics of these capacitation-associated protein reorganizations are not well characterized, it is apparent that dynamic sperm constituent proteins [23] and their interactomes [24,25] are contingent on the functional status of sperm. We therefore hypothesized that the capacitation-associated re-localization of tACE from the acrosomal to post-acrosomal region [13] enables tACE to interact with a novel cohort of sperm proteins to accomplish specific functions during capacitation and fertilization. In the current study, immunoprecipitation coupled with mass spectrometry was used to characterize the dynamic interactome of tACE in fresh versus heparin-capacitated bull sperm. Although many constituents of the tACE interactome remained unchanged, the relative abundances of specific proteins varied between fresh and capacitated sperm. In addition, we identified tACE interactome-constituent proteins recruited exclusively after capacitation. We inferred that compositional differences between fresh and capacitated sperm tACE interactomes have a functional role in capacitation and other downstream events that are prerequisite to fertilization. 

## 2. Materials and Methods

### 2.1. Semen Collection and Preparation

Fresh ejaculates from three mature Holstein bulls were obtained from a local artificial insemination center (Alta Genetics, Rocky View County, AB, Canada). Morphologically normal sperm samples with at least 70% progressive motility were used. Selected samples were immediately diluted 1:1 in TALPH [26] and transported in a thermos maintained at 37 °C. Semen samples were washed in Percoll gradients (90 and 45%) by centrifugation at 700× *g* for 20 min to remove somatic cells and dead sperm. The resulting sperm pellet was re-suspended in TALPH and re-centrifuged at 500× *g* for 10 min to remove remaining Percoll.

### 2.2. Sperm Capacitation

Percoll-washed sperm preparations at a concentration of 40 × 10^6^ sperm/mL were incubated in Sp-TALP on ice as a 0 h control [26], or in Sp-TALP supplemented with 10 μg/mL of heparin for 4 h at 39 °C and 5% CO_2_ in humidified air as the capacitation group. Our previous study demonstrated that incubation alone promotes tyrosine phosphorylation, to a lesser extent compared to capacitation groups [13]. An incubation control without a capacitating agent was not included within this experimental design due to logistical limitations with the number of samples. Incubated sperm were then evaluated for their content of tyrosine phosphoproteins through immunoblotting, their ability to undergo an acrosome reaction using the acrosome reaction (AR) test, and hyperactivation using Computer Assisted Sperm Analysis (CASA), as described [13].

### 2.3. Immunoprecipitation

Triton X-100-soluble fixed protein extracts were immunoprecipitated from fresh and 4 h capacitated sperm. Protein G beads stored in ethanol were first washed in protein extraction buffer (1% Triton X-100 in PBS) by centrifugation at 500× *g* for 30 s. Cross-linking was performed using tACE antibody (3 µg/mL) incubated with the protein-G bead slurry for 1 h at 4 °C on a rocker, in accordance with manufacturer’s instructions (Thermo Fisher Scientific, Missisauga, ON, Canada). The protein G beads were then conjugated with anti-tACE antibody (bovine anti-tACE immunoserum developed in rabbits against C-terminal sequence of sACE produced in collaboration with Thermo Fisher Scientific Antibody Services, Rockford, IL and tested for specificity with peptide blocking and mass spectometry [13]) by incubating at 4 °C for 1 h, according to the manufacturer’s protocol. The conjugated antibody preparation was then centrifuged at 500× *g* for 30 s to collect unbound beads and the supernatant was subsequently discarded after three washes. 300 μL of Triton X-100-soluble protein extracts from fresh or capacitated sperm were incubated with protein G beads for 30 min at 4 °C on a rocker to eliminate proteins that could nonspecifically bound to protein G beads as described previously [24] These precleared protein extracts were incubated with tACE antibody-conjugated beads for 4 h and then washed with 1% Tween-20 in PBS. The tACE antibody interactome complex was eluted from the beads by boiling with sample buffer for 5 min and then used for immunoblotting.

### 2.4. Identification of tACE Interactome by Liquid Chromatography and Tandem Mass Spectrometry (LC-MS/MS) Analysis

Polyacrylamide gel-resolved proteins were subjected to 0.1% Coomassie Brilliant Blue stain (0.1% Coomassie Brilliant Blue/50% methanol/10% glacial acetic acid/40% water) and destained with 10% methanol and 40% glacial acetic acid in water [24]. Coomassie-stained gel bands were excised in ~1 mm^3^ pieces and placed in Milli-Q water. Samples were sent to the Southern Alberta Mass Spectrometry (SAMS) facility at the University of Calgary for LC-MS/MS analysis. The protocols for in-gel protein digestion, peptide extraction, nano LC-MS/MS analysis of the digest, and Mascot database search were performed by the SAMS Centre. Samples were reconstituted using a mixture of ammonium bicarbonate and acetonitrile (50:50 *v*/*v*) at 50 mM. Samples were then subjected to a 30 min dithiothreitol reduction at 56 °C, followed by alkylation using iodoacetamide for 30 min at room temperature in the dark. The product was then trypsin-digested at 37 °C for 16 h. Acidification of the product was done using a solution containing 60% acetonitrile and 10% trifluoroacetic acid in water. Samples were lyophilized and subsequently resuspended with 1% formic acid in water.

### 2.5. LC-MS/MS Analysis

Liquid chromatography and tandem mass spectrometry were performed on an Agilent 6550 iFunnel quadrupole time-of-flight (Q-TOF) mass spectrometer with an Agilent 1260 Infinity HPLC-Chip Cube Interface controlled with MassHunter B.05.00 for analysis of cleaved tryptic peptides, as described [24]. Briefly, the A1 and B1 solutions (2.9 and 97% acetonitrile in 0.1% formic acid, respectively) were used to load the samples with a Nano Pump capillary to generate a gradient for peptide elution. The tryptic peptide aliquots (1 µL) were then loaded into a C18 trap column in an Agilent Chip set to enrichment mode with 3% B1 solution run at a flow rate of 2.5 µL/min. The chip was subsequently switched to analysis mode and eluted peptides in a gradient of B1 solution were created using the Nano Pump at a flow rate of 0.3 µL/min. These eluted peptides were electrosprayed into the Q-TOF mass spectrometer in positive auto MS/MS mode. Precursor ions were identified with a mass-to-charge (*m/z*) ratio between 275 and 1700 and were acquired at a scan rate of 250 ms/spectrum. A selection of precursors with the greatest abundance values in each cycle were selected using inclusion criteria of a charge value >1 and an intensity value of 1000 counts with a peptidic isotopic model fragment by collision-induced dissociation (CID). Fragment ions were obtained at 333 ms/spectrum, with an *m/z* ratio ranging from 50 to 1700.

### 2.6. Raw Data Conversion

Data transformation was performed as previously described [24]. The LC-MS/MS files were quantified using Aligent MassHunter software (B.05.00) and translated to a Mascot Generic Format (MGF) file. The Mascot algorithm (Matrix Sciences v2.4) was used to search the UniProt database using the MGF file. Selected parameters for the database search included *Bos taurus* taxonomy, trypsin as the enzyme, 1 as the maximum number of missed cleavage sites, a fixed carbamidomethylation post-translational modification, a variable modification of methionine oxidation, and a 0.2 Da mass error tolerance. Selection criteria for further analysis of peptides included a Mascot expectation value of <0.05 in addition to a Mascot ion score of 23 and were validated with Scaffold version 4 (Proteome Software Inc., Portland, OR, USA). A >95% probability threshold was used for protein identification, with contaminants manually eliminated. A calculated Normalized Spectral Count (NSC) was assigned to each protein using the product of the number of assigned spectra and the average spectral count of all proteins, divided by the total protein spectral count in a sample (Table 1).

### 2.7. Gene Ontology and Interactome Analyses

Gene ontology (GO) reports were acquired using PANTHER v16.0 (http://www.pantherdb.org/; access date on 17 July 2021) through input of acquired UniProt accession numbers to categorize proteins associated with tACE in fresh and capacitated sperm, or exclusively after capacitation. Classifications were reported in the context of molecular functions and biological processes, in addition to PANTHER gene ontology-related pathways. GO terms sourced from UniProtKB (https://www.uniprot.org/uniprot/ access date on 17 July 2021) were used for proteins with no identified hits in the PANTHER database. A tACE interactome network was created using STRING v11.5 (https://string-db.org/ access date on 17 July 2021) to present a theoretical interaction mapping between identified protein constituents of the tACE interactome from UniProt accession number inputs specific to the *Bos taurus* database.

### 2.8. Immunodetection of ATP1A4 in the tACE Interactome

The tACE interactomes prepared from fresh and 4 h heparin-capacitated sperm samples as described above were centrifuged at 10,000× *g* for 5 min; resulting supernatants were electrophoresed using 10% SDS-PAGE gels and electro-transferred to a nitrocellulose membrane. Membranes were blocked with 3% *w*/*v* skim milk in TTBS (Tween 20-Tris-based saline) for 1 h while rocking and subsequently incubated with anti-ATP1A4 (Protein A, Affinity Purified, specificity ensured by peptide blocking, University of Calgary [27]; 3.5 μL in 10 mL TTBS) and anti-β-tubulin (1:10,000 in TTBS, Thermo Fisher Scientific) antibodies overnight at 4 °C while rocking. Following incubation with primary antibodies, membranes were washed three times in TTBS for 10 min and were then incubated with HRP-conjugated secondary antibody (1:4000 in TTBS) at room temperature for 1 h while rocking. After washing, immunoreactive bands were visualized by exposure of the membrane to X-ray film. Specificity of anti-tACE and anti-ATP1A4 antisera were confirmed previously [10,27].

### 2.9. Immunocytochemistry for Co-Localization of β-Tubulin and tACE

Samples from fresh sperm and 4 h capacitated sperm were added to poly L-lysine charged slides and fixed with 4% PFA for 15 min. Sperm were then permeabilized using 0.5% Triton-X-100 in PBS for 30 min and washed three times in PBS. Blocking was done with 5% normal goat serum (NGS) in PBS for 30 min at RT. After three PBS washes, slides were co-incubated overnight at 4 °C with monoclonal anti-β-tubulin primary antibody developed in mouse (1:200) in addition to either polyclonal rabbit anti-tACE or polyclonal rabbit anti-ATP1A4 (1:10) in 1% NGS and PBS. Following five PBS washes, slides were incubated with goat anti-mouse Alexa 488 and goat anti-rabbit Alexa 555 secondary antibodies (1:1000 in PBS) at RT for 1 h in the dark. After PBS washes, slides were mounted with Vectashield containing DAPI (Vector Laboratories, Burlingame, CA, USA). A Zeiss Axio Observer Z1 inverted phase contrast fluorescence microscope and Axiocam MRc5 with AxioVision version 4.8.1 were used to capture images using a 100× objective oil-immersion lens.

### 2.10. Statistical Analysis

Results are reported as mean ± SD unless otherwise indicated. Semen samples from three bulls as biological replicates were used and mean normalized spectral counts of elucidated constituent proteins from 0- and 4-h heparin-capacitated sperm were reported. Protein abundance values from fresh and capacitated samples were analyzed using a paired *t*-test. Statistical analyses were performed using R version 2019, with *p* < 0.05 being considered significant.

## 3. Results

### 3.1. Mass Spectrometry for Characterization of the tACE Interactome

Ten proteins were interacting with tACE in both fresh and capacitated bovine sperm (Table 1). In addition, four proteins were interacting with tACE exclusively after heparin-induced capacitation (Table 2).

Identified proteins and mean normalized spectral counts as a measure of association probabilities for fresh and capacitated sperm samples (n = 3) are outlined in Table 1 and Table 2. Identified constituent tACE-interacting proteins common to both fresh and capacitated sperm included: A-Kinase Anchoring Protein 4 (AKAP4), Outer Dense Fiber Protein 2 (ODF2), Serotransferrin (TF), A-Kinase Anchoring Protein 3 (AKAP3), Serum Albumin (ALB), Mitochondrial ATP Synthase Beta Subunit (ATP5F1B), Testis-Specific Isoform of Na^+^/K^+^-ATPase (ATP1A4), Hexokinase (HK1), Calcium-Binding Tyrosine Phosphorylation-Regulated Protein (CABYR), and Mitochondrial ATP Synthase Subunit Alpha (ATP5A1). Interaction probability scores between tACE and AKAP4 were reduced (*p* < 0.01) after capacitation, whereas association dynamics between tACE and other identified constituents were not different between fresh and capacitated sperm (Table 1).

Mitochondrial Aldehyde Dehydrogenase (ALDH2), Inactive Serine/Threonine-Protein Kinase (VRK3), Tubulin-beta 4B chain (TUBB4B), and Tubulin-alpha 8 chain (TUBA8) were identified proteins unique to the capacitated tACE interactome that were not associated with tACE in fresh sperm (Table 2). The association probability scores among all four of these novel tACE interactions were increased (*p* < 0.01) in heparin-capacitated sperm (4 h) relative to fresh samples (0 h).

### 3.2. Gene Ontological Characterization of the tACE Interactome

**Figure 1:**
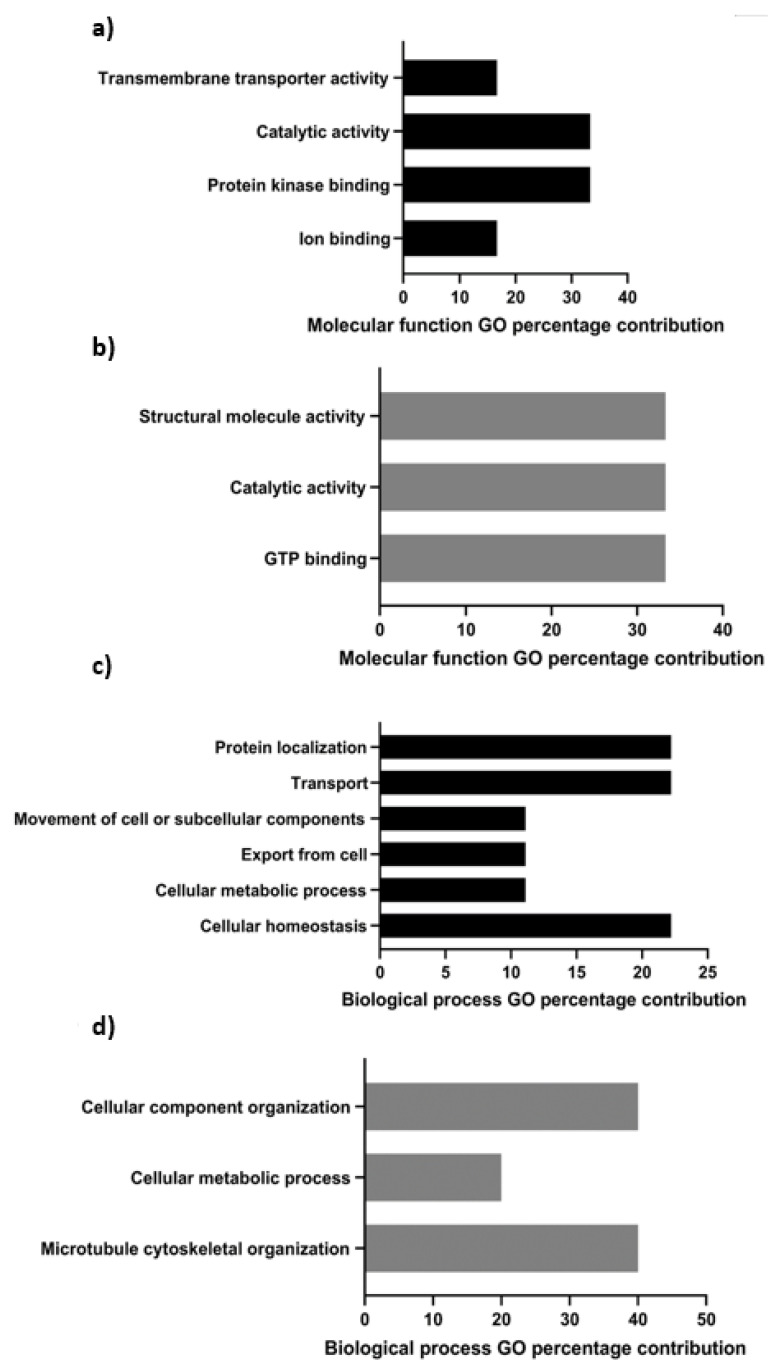

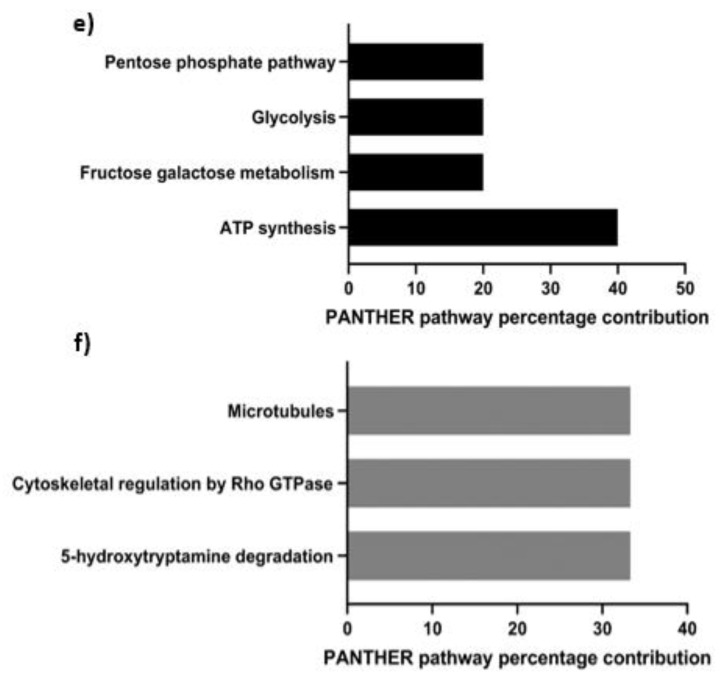
Percentage contributions of molecular function (**a**,**b**), biological process (**c**,**d**), and PANTHER ontological classification (**e**,**f**) pertaining to constituent interactome proteins associated with tACE in fresh and capacitated (**a**,**c**,**e**) or exclusively in capacitated (**b**,**d**,**f**) bovine sperm.

Based on PANTHER ontological analyses in conjunction with GO terms from UniProt for molecular function, protein kinase binding and catalytic activity were the major functional classifications for constituents of the tACE interactome present in both fresh and capacitated sperm, followed by transmembrane transporter activity and ion binding activity (Figure 1a). However, interactional classifications of these molecules with tACE remained similar between fresh and capacitated sperm. The tACE-interacting proteins identified exclusively in capacitated sperm were classified as being involved in structural molecule activity, catalytic activity, and GTP binding (Figure 1b).

Processes such as protein localization, transport, and homeostasis were identified biological functions (Figure 1c) of tACE interactomes present in both fresh and capacitated sperm, and interactions of these proteins remained similar between fresh and capacitated sperm. However, novel capacitation-induced binding partners were associated with cellular component organization, microtubule cytoskeletal organization, and cellular metabolic processes (Figure 1d).

PANTHER’s predicted pathway contributions for tACE interactome constituents common to fresh and capacitated sperm mainly pertained to catabolism through the pentose phosphate pathway, glycolysis, and fructose/galactose metabolism as processes leading to ATP synthesis (Figure 1e). The tACE interactome constituent proteins acquired exclusively after capacitation were involved in microtubule organization and cytoskeletal regulation by Rho GTPase, in addition to 5-hydroxytryptamine (5-HT) degradation (Figure 1f).

### 3.3. Interactive Network of Constituents of the tACE Interactome

**Figure 2:**
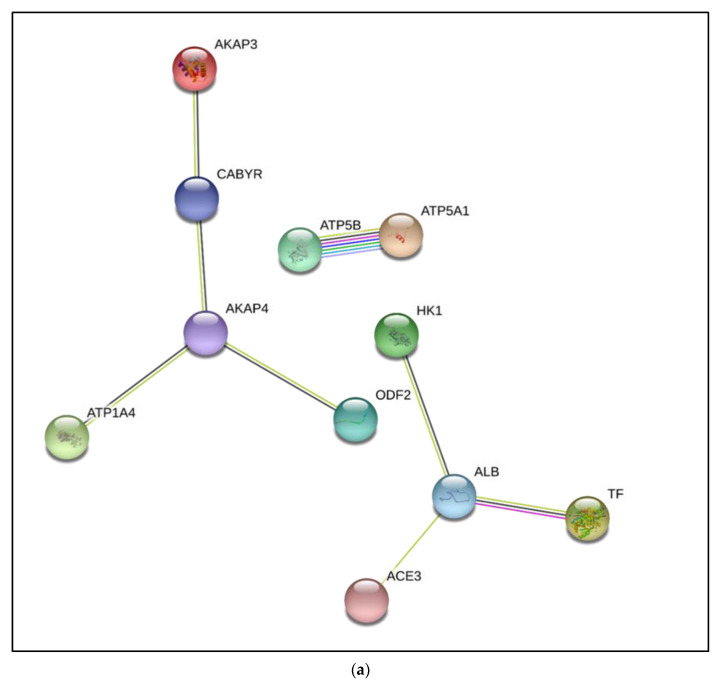

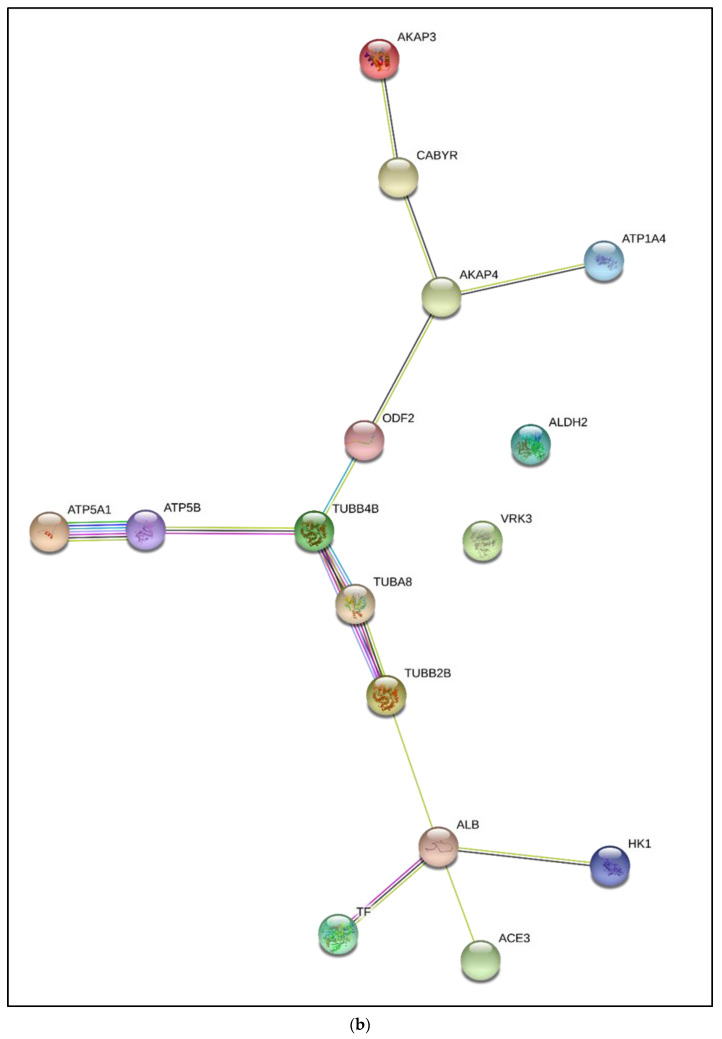

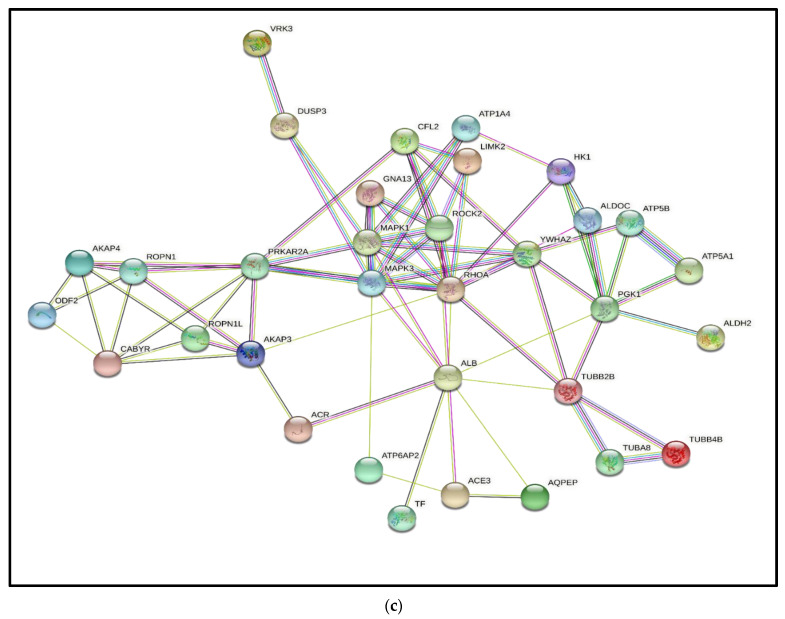
Protein–protein interaction (PPI) network of identified interactome constituents that remained associated with tACE (Testis Angiotensin Converting Enzyme) in fresh (**a**) and capacitated (**b**) bovine sperm, in addition to a comprehensive hypothetical interactional network (**c**). The network was generated through STRING v11.5 using *Bos taurus* taxonomy. Interactions are characterized by the number and color of interconnecting lines representing the strength and source of evidence of predicted associations with novel associations identified after capacitation that generated continuity within the network [PPI enrichment *p*-value in fresh sperm (**a**) = 0.000729; PPI enrichment *p*-value after capacitation (**b**) = 6.15 × 10^−6^; PPI enrichment *p*-value with presumed constituents elucidated from additional nodes to network to generate continuity (**c**) < 1.0 × 10^−16^]. AKAP3: A-Kinase Anchoring Protein 3; CABYR: Calcium-binding tyrosine phosphorylation-regulated protein; AKAP4: A-Kinase Anchoring Protein 4; ATP1A4: Sodium/potassium-transporting ATPase subunit alpha-4; ATP5B: ATP synthase F1 Subunit beta; ATP5A1: ATP synthase subunit alpha; ODF2: Outer dense fiber protein 2; HK1: Hexokinase 1; ALB: Albumin; TF: Transcription factor; ACE3: Angiotensin converting enzyme 3; ALDH2: Aldehyde dehydrogenase; VRK3: VRK Serine/Threonine Kinase 3; TUBB4B: Tubuin beta-4B chain; ATP5B: ATP synthase subunit beta; ATP5A1: ATP synthase subunit alpha; TUBA8: Tubulin alpha-8 chain; TUBB2B: Tubulin beta 2B; DUSP3: Dual specificity protein phosphatase 3; ROPN1: Rhophilin Associated Tail Protein 1; ROPN1L: Rhophilin associated tail protein 1 like; CFL2: Cofilin 2; GNA13: Guanine nucleotide-binding protein subunit alpha-13; PRKAR2A: cAMP-dependent protein kinase type II-alpha regulatory subunit; GNA13: Guanine nucleotide-binding protein subunit alpha-13; LIMK2: LIM domain kinase 2; ROCK2: Rho associated coiled-coil containing protein kinase 2; RHOA: Transforming protein RhoA; YWHAZ:14-3-3 protein zeta/delta (14-3-3ζ); PGK1: Phosphoglycerate kinase 1; AQPEP: Aminopeptidase Q.

The generated protein–protein interactional network of tACE interactome constituents that remained associated with tACE in fresh and capacitated sperm had significantly more interactions relative to a random protein set of similar size from the *Bos taurus* genome with a PPI enrichment *p*-value = 0.000729 (Figure 2a). Within generated networks (Figure 2a–c), ACE3 was presumed to be tACE for STRING accession input as a testis-specific homologue of ACE [28]. For the generated network of all identified tACE interactome constituents after heparin-induced capacitation (Figure 2b) with the inclusion of proteins identified exclusively after capacitation, there were significantly more interactions relative to a random protein set of similar size from the *Bos taurus* genome with a PPI enrichment *p*-value = 6.15 × 10^−6^. Contrary to our mass spectrometry results, ALDH2 and VRK3 were not predicted to interact within the STRING-generated network after capacitation based on proteins identified within our analysis. However, extrapolation from the STRING database of predicted interactions in addition to reports of the bovine sperm proteome generates continuity within the interactome and demonstrates the proteins through which tACE is predicted to interact (Figure 2c).

### 3.4. Immunolocalization of Constituents of the tACE Interactome

**Figure 3:**
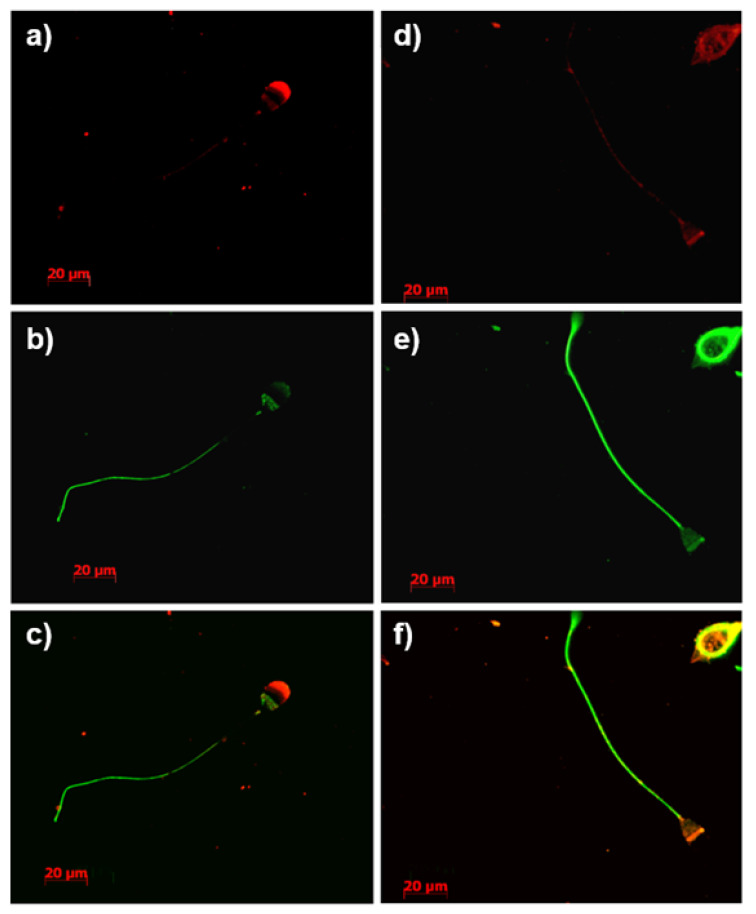
Immunolocalization of tACE (**a**,**d**; red), β-tubulin (**b**,**e**; green), with overlaid merges of red and green channels (**c**,**f**) in fresh (0 h control) (**a**–**c**) and capacitated (**d**–**f**) bovine sperm. Images were captured using an inverted phase contrast fluorescence microscope (Zeiss Axio Observer Z1) equipped with Axiocam MRc5 using a 100× oil-immersion objective lens. Co-localization of beta-tubulin and tACE within the sperm tail was demonstrated in Figure S1 as this was not clear in Figure 3.

**Figure 4:**
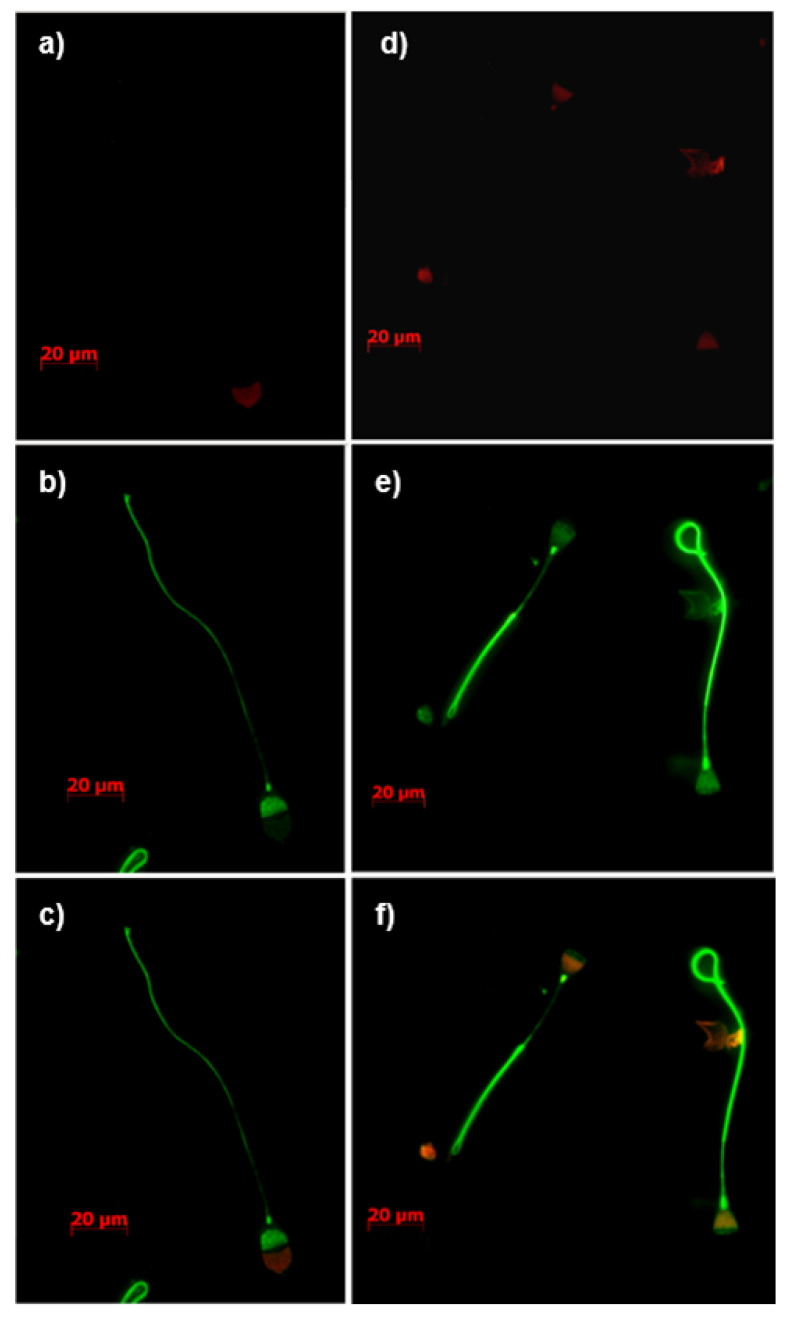
Immunolocalization of ATP1A4 (**a**,**d**; red), β-tubulin (**b**,**e**; green), with overlaid merges of red and green channels (**c**,**f**) in fresh (0 h control) (**a**–**c**) and capacitated (**d**–**f**) bovine sperm. Images were captured using an inverted phase contrast fluorescence microscope (Zeiss Axio Observer Z1) equipped with Axiocam MRc5 using a 100× oil-immersion objective lens. The secondary antibody controls (incubation without anti-tACE, anti-β-tubulin, and anti-ATP1A4) with associated phase contrast images were shown in Figure S2.

ATP1A4 and β-tubulin were selected as candidate proteins for validation of mass spectrometry results. Furthermore, tACE (red fluorescence) was localized to the acrosome, whereas β-tubulin (green fluorescence) was localized to the post-acrosomal region and the tail region in fresh (0 h) bovine sperm (Figure 3a–c). Upon 4 h heparin-induced capacitation, fluorescence intensity for tACE and β-tubulin was mainly co-localized to the post-acrosomal region and equatorial band of bovine sperm (Figure 3d–f). The β-tubulin fluorescence in the tail region remained unchanged before and after capacitation. Similarly, ATP1A4 was mainly localized to the acrosome in fresh sperm, whereas β-tubulin was localized to the post-acrosomal region in fresh sperm (Figure 4a–c). ATP1A4 (red fluorescence) subsequently localized to the post-acrosomal region in 4 h capacitated sperm (Figure 4d–f), where co-localization of β-tubulin (green fluorescence) and ATP1A4 was detected.

### 3.5. Immunodetection of Identified Constituents of the tACE Interactome

**Figure 5:**
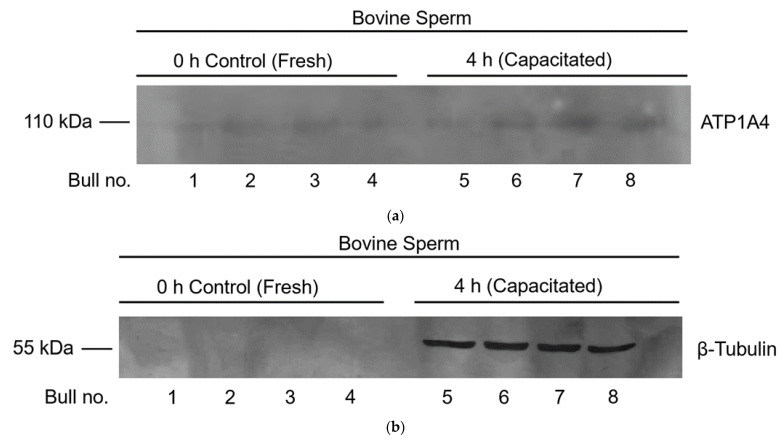
Co-immunoprecipitation data demonstrating interactions of tACE and ATP1A4 (**a**) and tACE and β-tubulin (**b**) in fresh and capacitated bovine sperm. Protein extracts from fresh and capacitated sperm were immunoprecipitated using anti-tACE antibody and immunodetected with anti-ATP1A4 (**a**) or anti-β-tubulin (**b**) antibody. Original unedited blots used in this figure were provided in Figure S3.

Mass spectrometry demonstrated that existing associations between tACE and ATP1A4 were not significantly different in fresh versus capacitated sperm (Table 1). Immunoprecipitation of sperm proteins from fresh and capacitated sperm using anti-tACE antibody and subsequent immunodetection of ATP1A4 from these immunoprecipitates further confirmed these results (Figure 5a). Interactions between tACE and β-tubulin were only observed after 4 h capacitation, as evidenced by mass spectrometry data (Table 2); these results were further confirmed with the presence of β-tubulin in tACE-immunoprecipitated proteins exclusively from the capacitated group (Figure 5b).

## 4. Discussion

The objective was to characterize differences between the tACE interactome in fresh and heparin-capacitated sperm. Although interactions between tACE and several identified proteins (ODF2, TF, AKAP3, ALB, ATP5F1B, ATP1A4, HK1, CABYR, and ATP5A1) remained unchanged in fresh versus capacitated sperm, interactions between tACE and AKAP4 were significantly decreased after capacitation. However, ALDH2, VRK3, TUBB4B, and TUBA8 exclusively interacted with tACE during capacitation, with implications for cytoskeletal and membrane reorganization, vesicle-mediated transport, GTP-binding, and redox regulation. Despite tACE function being integral to capacitation and fertility [11,12,13], the relevance of these interactions during capacitation remains to be elucidated.

In a previous study, we immunolocalized tACE to the acrosomal and principal piece regions of fresh bovine sperm [13]. Although capacitation did not influence location of tACE in the sperm tail, this protein was redistributed from the acrosomal to post-acrosomal region during capacitation [13]. The precursor to AKAP4 (pro-AKAP4) is initially localized to the periacrosomal membrane in boar sperm [29], whereas the mature protein is a component of the fibrous sheath [30,31]. Therefore, colocalization of tACE and AKAP4 in the sperm fibrous sheath may function in organization of target molecules to specific sperm compartments for signaling network scaffolding during capacitation. As AKAP4 is an ERK substrate and anchors Protein Kinase A (PKA), it is noteworthy that the association of PKA to AKAPs is necessary for sperm capacitation [31,32], and that the cAMP/PKA/AKAP4 pathway is a regulator of capacitation and the acrosome reaction [31]. Our LC-MS/MS results demonstrated a significant decrease in the interaction between tACE and AKAP4 after capacitation suggesting degradation of AKAPs during capacitation [33]. However, our STRING analysis identified an indirect association between these proteins after capacitation, in which tubulins may act as scaffolding proteins in concert with CABYR and AKAP3 [34,35,36] in addition to ODF2 [37,38,39,40] to complete the functional network. Although interaction among these proteins was reduced after capacitation, it is likely that capacitation-associated recruitment and redistribution of proteins preserved some vital interactions among these proteins after capacitation, allowing us to create the network of interaction.

Given that we reported the correlation of bull sperm characteristics with the content of tACE, Hexokinase-1, and ATP1A4 [27], associations between these protein networks in fresh versus capacitated sperm are relevant. Hexokinase is situated in close proximity to the plasma membrane within the fibrous sheath of murine sperm [41] and acrosome, mid-piece, and tail of human sperm [42]. This protein may supply ATP necessary for ATP1A4-mediated ion exchange across the plasma membrane [24,41]. The tyrosine-phosphorylated p95 Hexokinase isoform is present exclusively in the sperm head and testes, functionally resembling an integral membrane protein [43,44,45]. Although this isoform did not influence oocyte binding or acrosome reaction initiation in murine sperm [46], its presence within the sperm head may be relevant to tACE and ATPase function.

The ATP1A4 protein has wide implications for the regulation of motility and capacitation [47]. We demonstrated localization of ATP1A4 in the acrosomal region in fresh bovine sperm and its redistribution to the equatorial segment and post-acrosomal region during ouabain- [22,24] and heparin-induced capacitation in the current study. A comparable capacitation-associated redistribution of tACE from the acrosome to post-acrosomal region is also demonstrated in the current study. We previously reported de novo synthesis of ATP1A4 [9] which may contribute to the content, localization, and activity of this protein in the post-acrosomal region of capacitated sperm [22,47,48,49]. tACE has been identified as an IZUMO-interacting protein [50], and is thereby potentially involved in sperm–oocyte fusion. Similarly, our previous study suggested that ATP1A4 interacts with proteins involved in cell adhesion [24]. Therefore, an interaction between tACE and ATP1A4 suggests novel mechanisms by which these proteins contribute to sperm–oocyte interactions, warranting further research. It is noteworthy that our mass spectrometry data demonstrated an interaction between tACE and ATP1A4 before capacitation. However, our STRING analysis demonstrated an interaction between these proteins only after capacitation. It is likely that capacitation-associated redistribution of several proteins and de novo synthesis of ATP1A4 as demonstrated in our previous studies [24] may facilitate interaction of these proteins after capacitation. It is possible that a novel scaffolding protein (not identified in mass spectrometry analysis) connects these two proteins before capacitation and they associate through only TUBB2B after capacitation. Figure 4 demonstrates the interaction between ATP1A4 and beta-tubulin.

Our LC-MS/MS results identified four tACE interactome constituents unique to capacitated sperm. The ALDH2 protein may indirectly contribute to progressive motility of sperm by eliminating electrophilic aldehyde products [51]. As the post-acrosomal region and principal piece of sperm are vulnerable to electrophilic aldehyde adduction due to a deficient ALDH2 defense mechanism [51], perhaps the association of ALDH2 with tACE and its localization within these regions is in preparation for capacitation-associated hyperactivated motility and sperm–oocyte interaction. The function of ALDH2-mediated electrophilic product elimination may be coupled to mitochondrial ROS production that stimulates plasma membrane lipid peroxidation [52] and production of electrophilic aldehydes [53]. As endogenous ROS production is an important signaling mechanism for mammalian sperm capacitation and function, mediators such as ALDH2 may function to attenuate harmful by-products created by threshold ROS production [54]. However, contrary to our mass spectrometry results, our STRING analysis revealed no predicted associations between ALDH2 and the tACE interactome network within the *Bos taurus* taxonomy after capacitation potentially due to paucity of reports on the functional interactions between ALDH2 and tACE.

Similarly, STRING analysis did not reveal a direct association of VRK3 with the tACE interactive protein network in the *Bos taurus* taxonomy despite our LC-MS/MS identification of VRK3 as an interactome constituent with tACE exclusively after capacitation. The VRKs are catalytically inactive scaffold proteins within sperm [55,56] that may be involved in the inhibition of the extracellular signal-related kinase (ERK) pathway via activation of vaccinia H1-related (VHR) phosphatase [57,58]. The interaction between VRK3 and ERK and the associated inhibition of ERK has been reported in murine testicular tissue [58]. Although ERK molecules were not identified in our LC-MS/MS analysis, ERK may be involved in the interaction between VRK and the tACE interactome. Since the ERK pathway is involved in induction of tyrosine phosphorylation and capacitation [59,60], the interaction among VRK3, ERK and tACE may suggest regulation of the induction of the acrosome reaction in capacitated sperm [31,59].

The caveat to the capacitated STRING network is that the TUBB2B, a protein not identified in our mass spectrometry results but previously reported in sperm and implied to be a constituent of the beta-tubulin cytoskeletal complex [61,62,63,64], was included in our analysis to generate continuity within the protein–protein interaction network. This addition was to illustrate that tubulins act as a scaffold through which the three independent segregated networks in fresh sperm interconnect upon heparin-induced capacitation. Localization of tubulin proteins within the post-acrosomal region in various mammalian sperm may have a role in capacitation, the acrosome reaction, and fertilization through its interactions and spatial associations with other cytoskeletal proteins [65,66]. Furthermore, distributional irregularities of beta-tubulin within the sperm head have been associated with infertility [65]. In the current study, TUBB4B and TUBA8 were constituents of the tACE interactome exclusively in capacitated sperm. Given the heterogeneity of tubulin subunits, it is likely that other isoforms such as TUBB2B were also present in sperm [23,63]. Although only two isoforms were identified from the LC-MS/MS output, results of our protein–protein interaction network demonstrated additional tubulin isoforms such as TUBB2B may act as scaffolding proteins through which the tACE interactome network integrates with associated constituents upon capacitation. Beta-tubulin epitopes have been immunolocalized to the equatorial [65,67] and post-acrosomal region [65,68] in mammalian sperm, with distributional changes between normal and abnormal human sperm, suggesting a potential role in sperm function. The functional relevance of these interactions formed exclusively after capacitation between tubulin proteins and the tACE interactome may be to spatially organize the sperm plasma membrane and associated proteins to orchestrate downstream events such as an acrosome reaction. This hypothesis is supported by the redistribution of tubulins in the head of human and rodent sperm to the equatorial segment and post-acrosomal region after an acrosome reaction, which may be involved in membrane remodeling prior to sperm–oocyte fusion [66].

Our immunoprecipitation and mass spectrometry results characterizing the tACE interactome were supported by a study characterizing the Rho-A interactome [69] in which ACE, AKAP4, ODF2, hexokinase, and alpha- and beta-tubulin were identified constituent proteins of bovine caudal epididymal sperm.

Regardless, the current status of the proposed tACE interactome was not comprehensive, due to limitations with the assay and analysis of interaction probability scores. Proteins such as Rho-A in addition to cohorts of tubulin isoforms were not identified within our LC-MS/MS output, despite the report of tACE as a constituent of the Rho-A interactome which is suggested to mediate actin cytoskeletal reorganizations preceding the acrosome reaction [69]. As a result, proteomic results from RHOA-interacting constituents identified in bovine caudal spermatozoa [69] in addition to STRINGs predicted associations were used to generate a more comprehensive theoretical interactome in which RHOA-interacting proteins including cAMP-dependent protein kinase type II-alpha regulatory subunit (PRKAR2A), ropporin (ROPN1), YWHAZ, phosphoglycerate kinase 1 (PGK1), and MAPK proteins which may scaffold the constituents identified in our LC-MS/MS analysis before capacitation, in addition to integrating ALDH2, VRK3, TUBB4B, and TUBA8 after capacitation.

Taken together with our results, the tACE interactome constituents may vary temporally, based on the functional status of sperm. The functional breadth of the tACE interactive complex and its capacitation-associated dynamics emphasized the importance of tACE in the regulation of sperm function.

## 5. Conclusions

The tACE interactome constituents varied depending on the functional status of sperm, suggesting that tACE is involved in the regulation of specific sperm functions by its dynamic and temporal associations with a multitude of proteins.

## Figures and Tables

**Table 1:** Mean normalized spectral counts (±SD) as a measure of quantified abundance of proteins interacting with tACE in fresh (0 h control) and capacitated bovine sperm (n = 3) using liquid chromatography and tandem mass spectrometry (a,b: *p* < 0.05).

Accession Number	Protein Name	Gene Name and GO Terms		Mean (±SD) Normalized Spectral Count in Fresh Sperm	Mean (±SD) Normalized Spectral Count in Capacitated Sperm
F1MYH5	A-Kinase anchoring protein 4	AKAP4	Protein binding; Protein localization; Scaffold/adaptor	307.14 ± 39.39 ^a^	205.90 ± 38.45 ^b^
Q2T9U2	Outer dense fiber protein 2	ODF2	Developmental protein; Spermatogenesis	141.85 ± 28.22	100 ± 26.32
G3X6N3	Serotransferrin	TF	Iron binding; Iron ion transport and localization; ERK1 and ERK2 cascade	120.30 ± 36.40	87.80 ± 15.18
F1MJS8	A-Kinase anchoring protein 3	AKAP3	Protein binding; Protein localization; Scaffold/adaptor	107.90 ± 19.37	73.38 ± 19.20
A0A140T897	Serum albumin	ALB	Chaperone binding; Zinc ion binding and localization	250.17 ± 29.10	195.32 ± 15.72
P00829	Mitochondrial ATP synthase beta subunit	ATP5F1B	ATPase activity; Mitochondrial transmembrane transport; Cellular metabolic process; ATP synthesis	26.26 ± 2.47	22.61 ± 5.90
E1B8N5	Testis-Specific isoform of Na^+^/K^+^-ATPase	ATP1A4	ATPase activity; Ion homeostasis and localization	25.85 ± 4.37	22.82 ± 6.5
P27595	Hexokinase	HK1	Transferase activity; ATP binding; Glucose homeostasis; Pentose phosphate pathway	80.57 ± 17.16	55.14 ± 10.31
Q32L61	Calcium binding tyrosine phosphorylation regulated	CABYR	Calcium ion binding; Sperm capacitation	30.10 ± 4.22	28.16 ± 3.90
P19483	Mitochondrial ATP synthase subunit α	ATP5A1	ATP synthase activity; Cellular metabolic process	15.12 ± 4.40	10.69 ± 3.13

**Table 2:** Mean normalized spectral counts (±SD) as a measure of quantified abundance proteins interacting with tACE exclusively identified in capacitated bovine sperm (n = 3) using liquid chromatography and tandem mass spectrometry.

Accession Number	Protein Name	Gene Name GO Terms		Mean (±SD) Normalized Spectral Count in Fresh Sperm	Mean (±SD) Normalized Spectral Count in Capacitated Sperm
P20000	Aldehyde dehydrogenase, mitochondrial	ALDH2	Oxidoreductase; NAD binding; 5-Hydroxytryptamine degradation	0 ± 0	15.12 ± 3.66
Q2YDN8	Inactive serine/threonine-protein kinase	VRK3	Protein phosphorylation; Vesicle-mediated transport signal transduction; Membrane organization	0 ± 0	10.10 ± 3.45
Q3MHM5	Tubulin-beta 4B chain	TUBB4B	Cytoskeleton; Microtubule cytoskeletal organization; Cytoskeletal regulation by Rho GTPase	0 ± 0	150.90 ± 20.13
Q2HJB8	Tubulin-alpha 8 chain	TUBA8	Cytoskeleton; Microtubule cytoskeletal organization	0 ± 0	80.92 ± 19.84

## Supplementary Materials

The following are available online at https://www.mdpi.com/article/10.3390/cimb44010031/s1, Figure S1: Immunofluorescent localization of anti-tACE (a) and anti-β-tubulin (b), with overlaid merge of channels (c) and DAPI nuclear stain (d) in 4 h heparin-capacitated bovine sperm. Figure S2: Secondary antibody control and associated phase contrast field of tACE (a,b), β-tubulin (c,d), and ATP1A4 (e,f) in fresh (c–f) and 4 h capacitated (a,b) bovine sperm. Figure S3: Original unedited blots used in Figure 5 of the manuscript for (a) ATP1A4 and (b) β-tubulin. An additional blot (c) for β-tubulin immunoprecipitated using anti-tACE antibody exclusively after capacitation is also shown.
