# Supplementary material for: Characterization of the Testis-Specific Angiotensin Converting Enzyme (tACE)-Interactome during Bovine Sperm Capacitation
Source: Curr Issues Mol Biol. 2022 Jan 17;44(1):449–69. doi: 10.3390/cimb44010031 (PMC8928970; doi:10.3390/cimb44010031)
Supplement: Supplementary file 1 [file cimb-44-00031-s001.zip › cimb-1506345-supplementary.pdf]

Supplementary materials: Characterization of the testis-specific Angiotensin Converting Enzyme (tACE)-interactome during bovine sperm capacitation

Mina Ojaghi, Jacob Varghese, John P. Kastelic, and Jacob C. Thundathil*

Description on tACE immunolocalization

The purpose of Figure 3 was to demonstrate the difference in tACE and beta-tubulin distribution before and after capacitation within the sperm head. We previously reported immunolocalization of tACE within the sperm head and sperm tail [13]. Figure S1 demonstrates co-localization of beta-tubulin and tACE within the sperm tail during the current study (this was not clear in Figure 3), captured using a spectral confocal microscope (Leica TCS SP8) using a 63x objective lens immersed in oil.

The secondary antibody controls (incubation without anti-tACE, anti-β-tubulin, and anti-ATP1A4) with associated phase contrast images are included in Figure S2. A lower magnification (20x) was used to demonstrate that background fluorescence due to non-specific binding was negligible in a larger field of view.

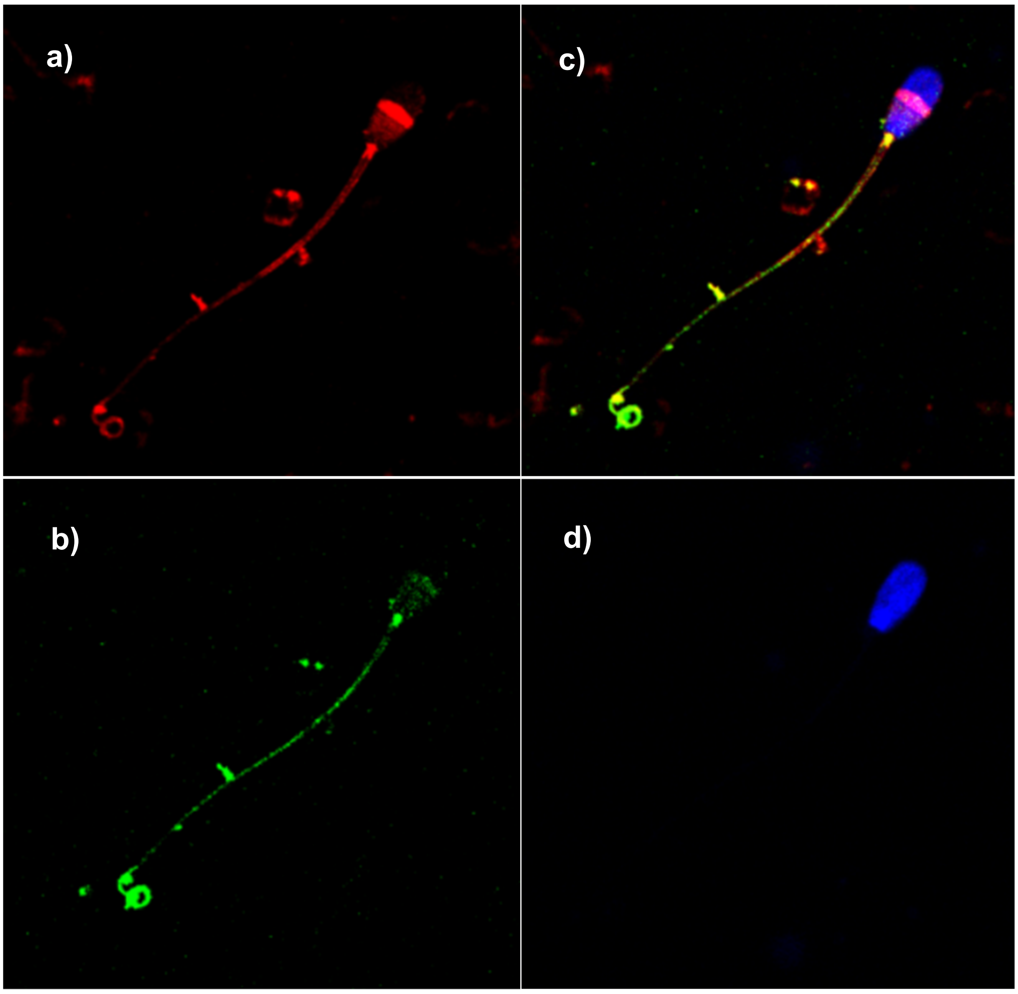

Figure S1: Immunofluorescent localization of anti-tACE **(a)** and anti-β-tubulin **(b)**, with overlaid merge of channels **(c)** and DAPI nuclear stain **(d)** in 4 h heparin-capacitated bovine sperm. Images were captured using a spectral confocal microscope (Leica TCS SP8) using a 63x objective lens immersed in oil.

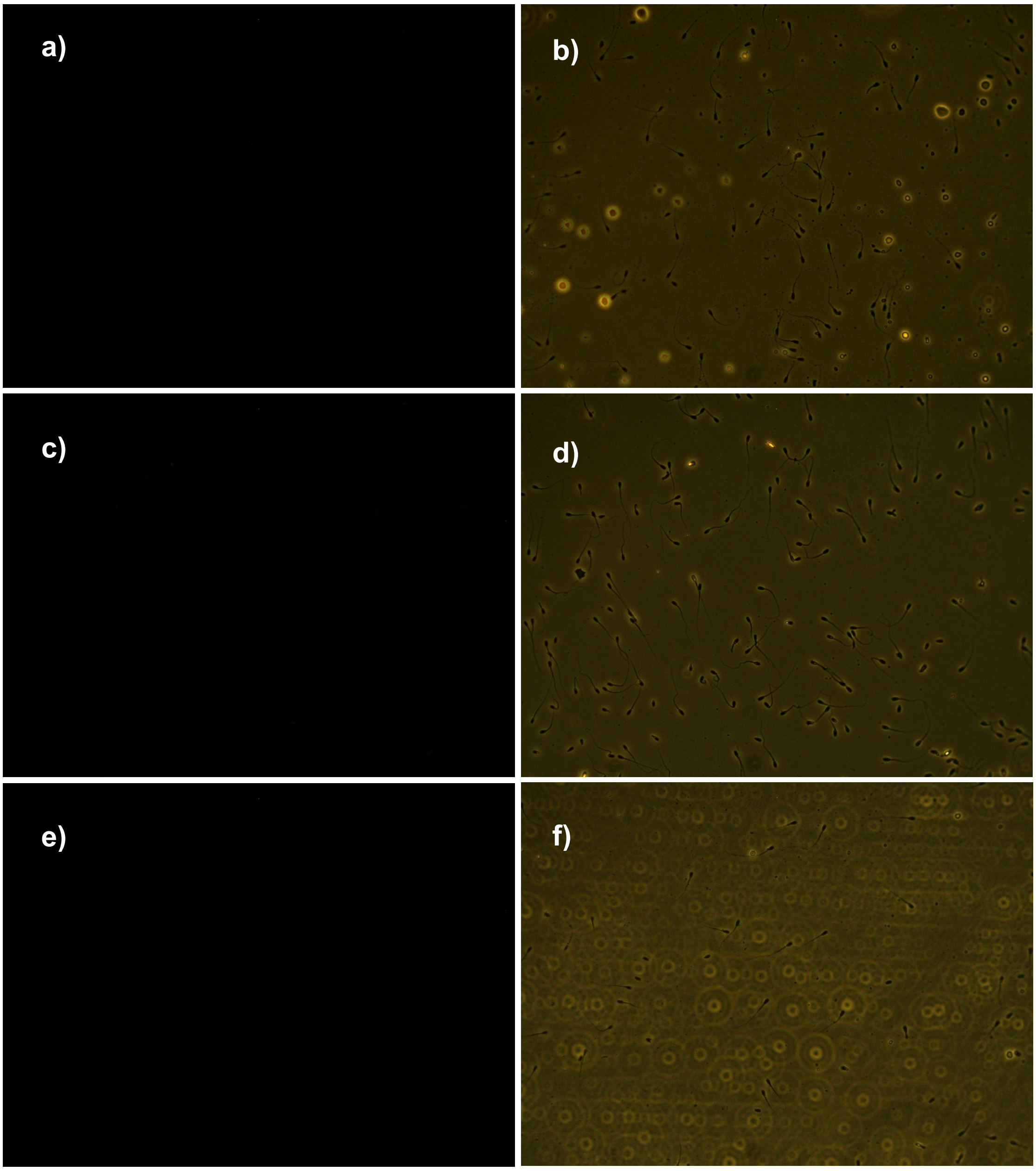

Figure S2: Secondary antibody control and associated phase contrast field of tACE **(a,b)**, β-tubulin **(c,d)**, and ATP1A4 **(e,f)** in bovine sperm. Images were captured using an inverted phase contrast fluorescence microscope (Zeiss Axio Observer Z1) equipped with Axiocam MRc5 using a 20x objective lens.

Original unedited blots

In the supplemental section, we provided the original raw blots indicating the proteins probed after immunoprecipitation with tACE.

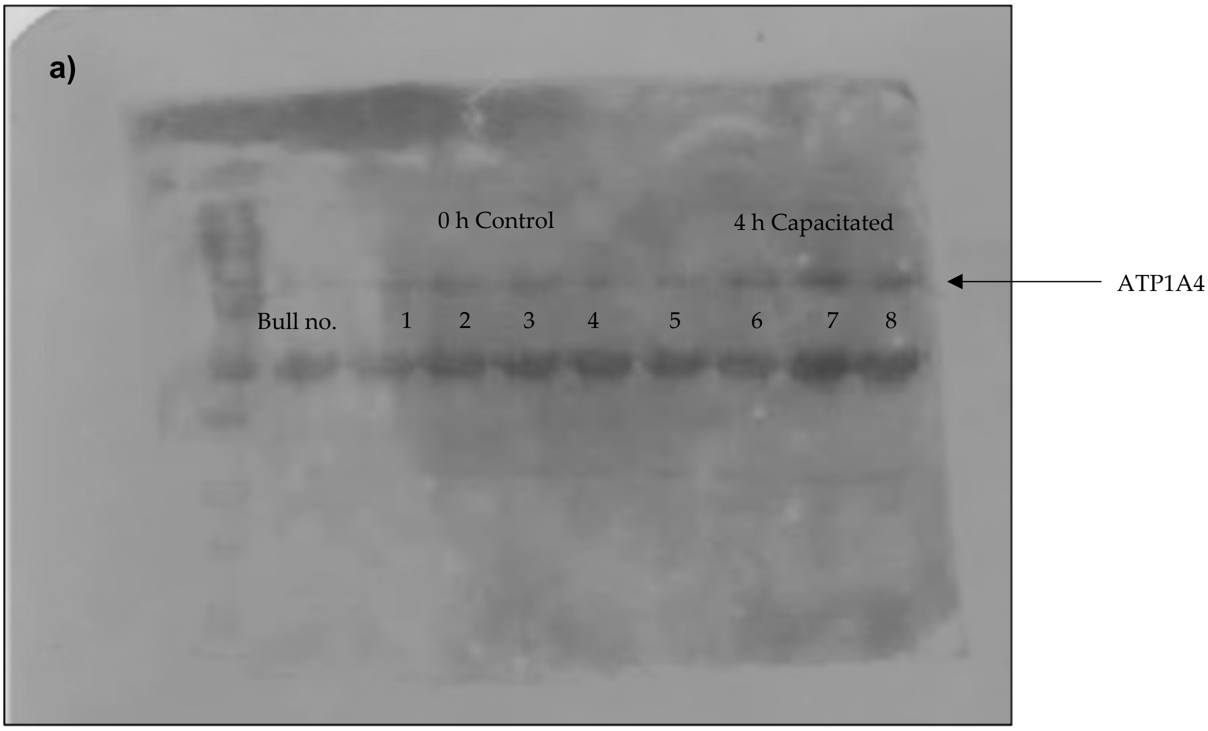

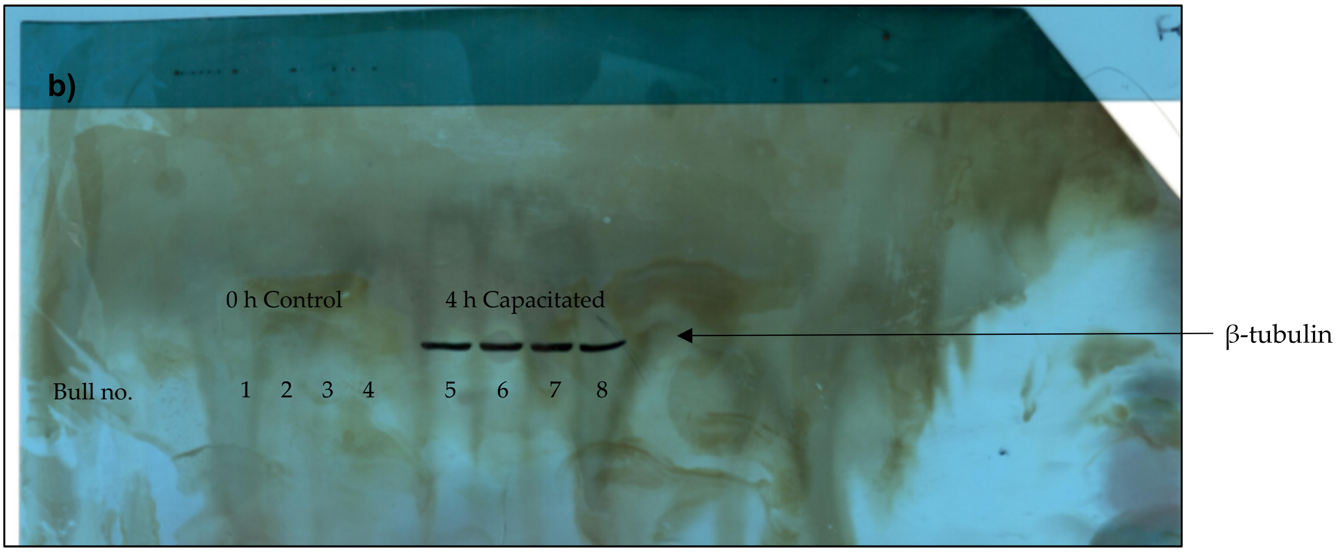

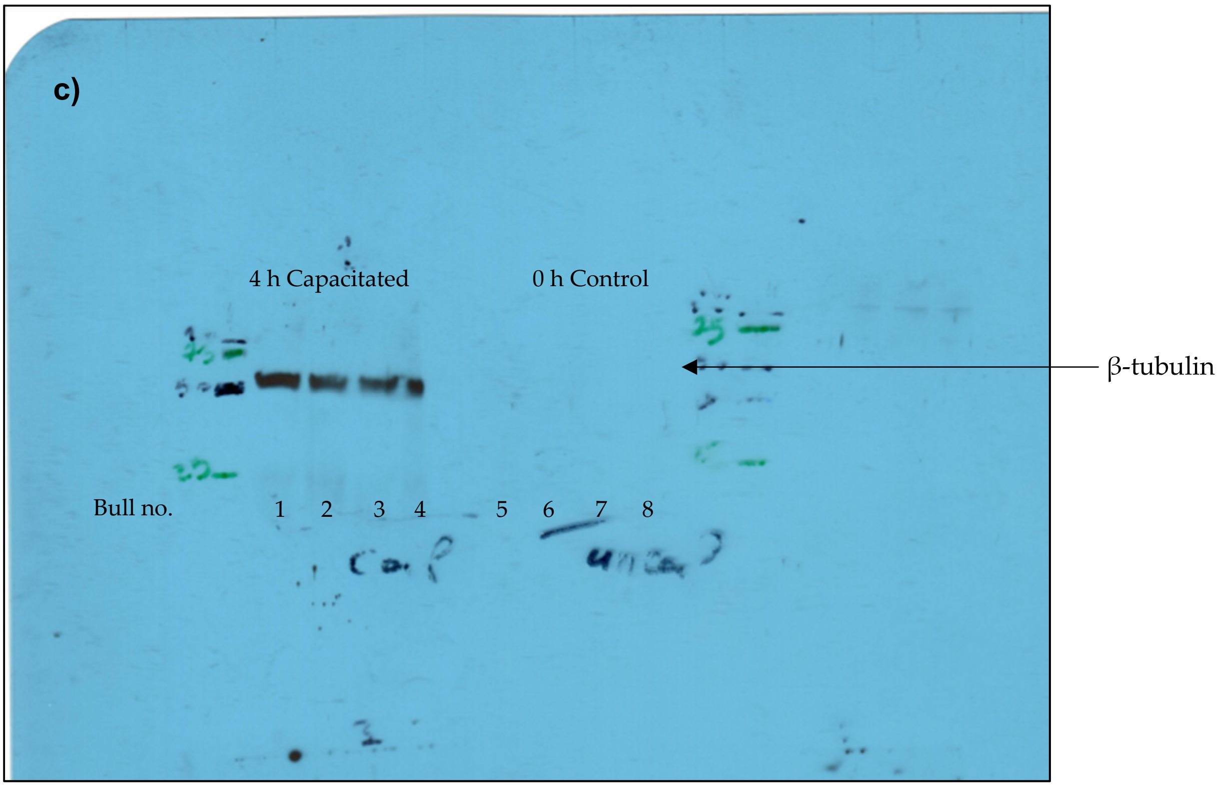

Figure S3: Original unedited blots used in Figure 5 of the manuscript for **(a)** ATP1A4 and **(b)** β-tubulin. An additional blot **(c)** for β-tubulin immunoprecipitated using anti-tACE antibody exclusively after capacitation is also shown.
